# Revisiting the Charge-Transfer States at Pentacene/C_60_ Interfaces with the GW/Bethe–Salpeter Equation Approach

**DOI:** 10.3390/ma13122728

**Published:** 2020-06-16

**Authors:** Takatoshi Fujita, Yoshifumi Noguchi, Takeo Hoshi

**Affiliations:** 1 Institute for Molecular Science, Okazaki, Aichi 444-0865, Japan; 2 Department of Applied Chemistry and Biochemical Engineering, Graduate School of Engineering, Shizuoka University, Hamamatsu, Shizuoka 432-8561, Japan; NOGUCHI.Yoshifumi@shizuoka.ac.jp; 3 Department of Applied Mathematics and Physics, Tottori University, Tottori 680-8550, Japan; takeo.hoshi.tottori.u@gmail.com

**Keywords:** charge-transfer states, donor/acceptor interfaces, organic photovoltaics, excited-state calculations, fragment molecular orbital method, GW+Bethe–Salpeter equation

## Abstract

Molecular orientations and interfacial morphologies have critical effects on the electronic states of donor/acceptor interfaces and thus on the performance of organic photovoltaic devices. In this study, we explore the energy levels and charge-transfer states at the organic donor/acceptor interfaces on the basis of the fragment-based GW and Bethe–Salpeter equation approach. The face-on and edge-on orientations of pentacene/C_60_ bilayer heterojunctions have employed as model systems. GW+Bethe–Salpeter equation calculations were performed for the local interface structures in the face-on and edge-on bilayer heterojunctions, which contain approximately 2000 atoms. Calculated energy levels and charge-transfer state absorption spectra are in reasonable agreements with those obtained from experimental measurements. We found that the dependence of the energy levels on interfacial morphology is predominantly determined by the electrostatic contribution of polarization energy, while the effects of induction contribution in the edge-on interface are similar to those in the face-on. Moreover, the delocalized charge-transfer states contribute to the main absorption peak in the edge-on interface, while the face-on interface features relatively localized charge-transfer states in the main absorption peak. The impact of the interfacial morphologies on the polarization and charge delocalization effects is analyzed in detail.

## 1. Introduction

The charge-transfer (CT) states across the interface between electron-donating and electron-accepting materials play central roles in the charge photogeneration process and the power conversion efficiency of organic photovoltaics [1,2,3]. The energy of the interfacial CT state correlates with the open-circuit voltage value [4]. The energy offset between the interfacial CT state and an exciton state provides the driving energy for charge separation, but constitutes energy loss in the open-circuit voltage [5]. The CT state can decay to the ground state by radiative or nonradiative charge recombination. In particular, the nonradiative recombination is responsible for a significant fraction of the energy loss in the open-circuit voltage [6]. Because of the central importance, understanding and engineering of CT states are essential for improving the power conversion efficiency of organic solar cells.

Ab initio quantum chemical calculations can provide detailed microscopic insights into interfacial CT states [7,8]. The CT states of organic donor/acceptor systems have been studies using time-dependent density functional theory (TDDFT) [9,10,11,12]. However, standard DFT calculations cannot describe the gap renormalization, i.e., the stablization of hole and electron transport levels in polarizable environments [13,14,15]. Recently, many-body perturbation theory within the GW approximation has been applied to organic materials [14,15,16,17,18,19,20,21]. In the GW method, the polarization induced by the charged excitation can be appropriately described in the self-energy via a spatially- and energy-dependent dielectric function [22]. In addition, excited-state calculations based on the Bethe–Salpeter equation (BSE) [23] within GW, denoted as GW+BSE, can offer the correct asymptotic behaviors of CT states with respect to the large electron–hole (e–h) separation [21,24] in both gas and solid phases. We have developed a large scale GW implementation that can handle a large molecular system containing over 1000 atoms [15,21]. Application of GW to a large system allows for unified descriptions of the polarization and delocalization effects in a disordered molecular system.

**Figure 1:**
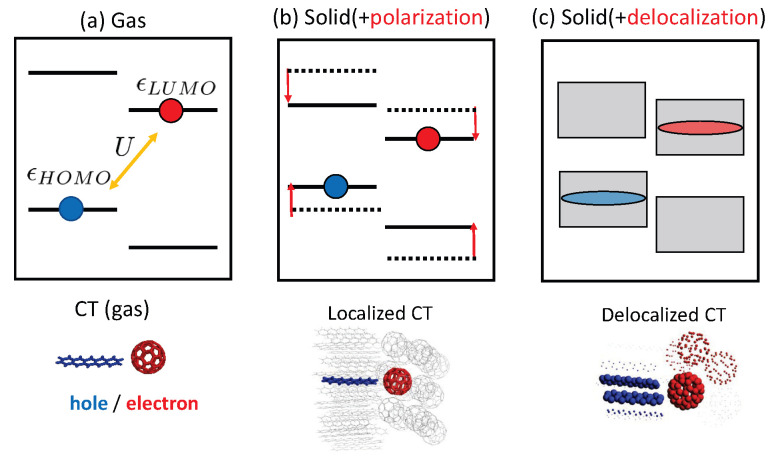
Schematics of solid-state effects on the interfacial CT states: (**a**) a CT state in a gas phase, (**b**) a localized CT state in a solid phase, and (**c**) a delocalized CT state in a solid phase.

In this study, we investigate the CT states in pentacene(PEN)/C_60_ interfaces as model systems and highlight the polarization and delocalization effects. The effects of polarization and delocalization on the interfacial CT states are schematically represented in Figure 1. In a gas phase (Figure 1a), the energy of the CT state is given by the highest-occupied molecular orbital (HOMO) energy of a donor molecule, the lowest-unoccupied molecular orbital (LUMO) energy of an acceptor molecule, and the Coulomb interaction between the electron and the hole. The solid-state polarization effects reduce the energy gap between the donor HOMO and the acceptor LUMO (Figure 1b) and weakens the e–h interaction. In a solid phase, the electron or hole wave function can be delocalized over multiple acceptor or donor molecules, and delocalized CT states can be formed (Figure 1c). This charge delocalization can further shift the energy gap and possibly weaken the e–h interaction. It is argued that the charge delocalization reduces the e–h interaction and thus allows for efficient charge separation [25,26,27,28].

We consider the edge-on (standing-up) and face-on (lying-down) orientations of PEN/C_60_ interfaces. The energy levels across PEN/C_60_ interfaces have been determined by the photoelectron spectroscopy studies [29,30,31]. The CT state absorption of PEN/C_60_ has been obtained by Brigeman et al. [32] on the basis of the polarized external quantum efficiency (EQE) measurements. Later, Lin et al. [33] have examined the dependence of the CT energies on the different compositions and morphologies of the interfaces. The excited states of PEN/C_60_ clusters have been calculated at the TDDFT level [10,11,12,34]. Verlaak et al. [35] have performed microelectrostatic calculations for the edge-on and the face-on PEN/C_60_ bilayer heterojunctions, pointing to the importance of molecular environments in their energy levels. We have also presented fragment-based excited state calculations for the PEN/C_60_ interfaces [15]. However, those excited state calculations were carried out at the configuration interaction single level with correction by TDDFT. In particular, the induction contribution of polarization energy could not be accurately described in our earlier calculation, resulting in the overestimation of charge-separated states relative to the short-range CT states. In this manuscript, we revisit the energy levels and CT states in the PEN/C_60_ interfaces by using GW+BSE method.

## 2. Methods

This section briefly summarizes computational details. Herein, we carried out all-electron calculations based on the fragment molecular orbital (FMO) method [36,37] to access the energy levels and CT states of the PEN/C_60_ interfaces. In the FMO method, an entire system is first divided into many small subsystems referred as fragments, and the total energy or physical properties are then approximated from self-consistent field (SCF) calculations for fragment monomers, dimers, and optionally trimers in the electrostatic embedding potentials from other fragments. If a molecule is assigned as an independent fragment in an FMO calculation, then the SCF calculation for a fragment monomer provides the one-electron orbitals localized within the molecule. Recent developments in the FMO method have allowed for calculations for delocalized orbitals [38,39] and for delocalized excited states [40]. We have developed the GW method within the FMO method which enables quasiparticle calculations for organic material systems [15,21].

**Figure 2:**
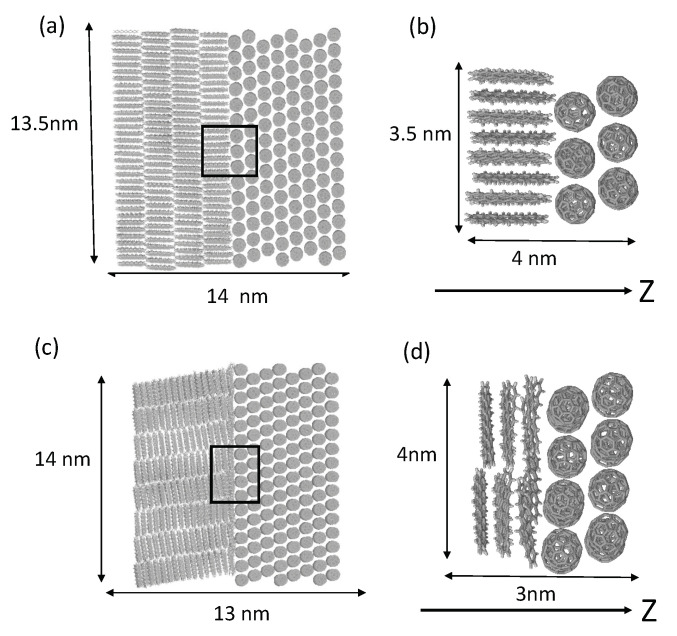
Atomistic structures of the (**a**) edge-on and (**b**) face-on orientations of the PEN/C_60_ bilayer heterojunctions. (**c**,**d**) The local interface structure treated quantum-mechanically by the excited-state method. Reproduced from ref. [41] with permission from the PCCP Owner Societies.

We employed the edge-on and the face-on orientations of PEN/C_60_ bilayer heterojunctions, as shown in Figure 2, as two limiting interface orientations. The details of the modeling and subsequent molecular dynamics simulations have been presented elsewhere [41]. The FMO calculations were performed for the local interface structures shown in Figure 2b,d, in which 36 PEN and 15 C_60_ molecules are included in the local edge-on interface (Figure 2b), while 24 PEN and 20 C_60_ molecules are included in the local face-on interface (Figure 2d). The remaining molecules in the total structure in Figure 2a,c were incorporated as external point charges. The FMO-GW calculations were performed with each molecule being assigned as an independent fragment. The B3LYP was used as a starting point for the one-shot FMO-GW calculation with 6-31G* basis set. Electronic structure calculations were performed using ABINIT-MP software [37,42,43].

Next, we consider the quasiparticle energies obtained from the FMO-GW and discuss how polarization energies are described in our calculations. FMO-GW calculations provide the quasiparticle energy for an molecular orbital (MO) localized within an *I*th molecule:

(1) $$\epsilon_{GW,p}^{I}=\epsilon_{p}^{I}+Z_{p}\left[\Sigma_{FMO-GW,p}^{I}-V_{p}^{XC}\right],$$

where $\epsilon_{p}^{I}$ is the Hartree-Fock (HF) or Kohn-Sham (KS) orbital energy, $Z_{p}$ is the renormalization factor, $V_{p}^{XC}$ is the exchange-correlation potential in the DFT, and *p* is an MO index. Here, the FMO-GW self-energy is given by

(2) $$\Sigma_{FMO-GW,p}^{I}=\Sigma_{GW^{I},p}^{I}+\Sigma_{COHSEX,p}^{I}-\Sigma_{COHSEX^{I},p}^{I}.$$

The computational details for these terms can be found in our previous study [21]. The quasiparticle energy ($\epsilon_{GW,p}^{I}$) of the *I*th molecule corresponds to the energy necessary to create an electron (*p* = LUMO) or a hole (*p* = HOMO) that is localized within the *I* molecule. The FMO-GW can appropriately describe the polarization energy, i.e., the MO energy change between an isolated molecule and a molecule in a solid phase. Recent studies have shown that polarization energy can be further divided into electrostatic and induction components [44,45,46]. The electrostatic component represents the interaction between the charge carrier and the permanent electrostatic multipole moments of surrounding molecules. The induction component denotes the interaction between the charge carrier and dipole moments of surrounding molecules that are induced by the formation of the charge carrier. The latter is also refereed as the electronic polarization [45,46], induced polarization [21], or dynamical polarization [47]. In the FMO-GW method, the electrostatic contribution is included in the reference fragment MO energies ($\epsilon_{p}^{I}$), which are obtained from an FMO calculation at the HF or DFT level. On the other hand, the induction contribution is incorporated in the FMO-GW quasiparticle energy through the Coulomb-hole plus screened exchange (COHSEX) self-energy term, $\Delta \Sigma_{COHSEX,p}^{I}=\Sigma_{COHSEX,p}^{I}-\Sigma_{COHSEX^{I},p}^{I}$. By analyzing these terms in the FMO-GW quasiparticle energy in detail, we can disentangle electrostatic and induction contributions of the polarization energy.

In a solid state, a one-electron wave function can be delocalized over multiple molecules by intermolecular electronic couplings. In FMO calculations, such the orbital delocalization effects can be treated by using the FMO-linear combination of molecular orbital (FMO-LCMO) method [38,39,48]. In the FMO-LCMO method, the one-electron Hamiltonian (Fock matrix) for an entire system is calculated on the basis of fragment monomer MOs and then is diagonalized to approximate the canonical (delocalized) orbitals of the entire system. The one-electron Hamiltonian in the FMO-LCMO method can be written in a tight-binding form by canonical transformation. For example, the unoccupied orbitals of an entire system, which represent the energy levels of a delocalized electron, can be obtained by the following Hamiltonian,

(3) $$H^{e}=\sum_{I}\epsilon_{GW,L}^{I}|\psi_{L}^{I}\rangle \langle \psi_{L}^{I}|+\sum_{I\neq J}t_{LL}^{IJ}|\psi_{L}^{I}\rangle \langle \psi_{L}^{I}|,$$

where $\psi_{L}^{I}$ is the LUMO of an *I*th molecule, and $t_{LL}^{IJ}$ is the LUMO–LUMO transfer integral between *I*th and *J*th molecules. Correspondingly, the energy levels for a delocalized hole are calculated by the Hamiltonian composed of HOMOs. In this study, the off-diagonal elements between the occupied and unoccupied orbitals were neglected, and thus occupied-occupied ($H^{h}$) and unoccupied-unoccupied ($H^{e}$) blocks of the Hamiltonian were separately treated. In our calculations, the MO energies were obtained at the FMO-GW level, whereas the transfer integrals were calculated at the FMO-B3LYP level [49]. Three occupied monomer MOs per fragment were included in $H^{h}$, while five unoccupied monomer MOs per fragment were included in $H^{e}$.

As depicted in Figure 1b, a localized CT state consists of a localized electron in an acceptor molecule and a localized hole in a donor molecule. Assuming the intermolecular CT state can be well described by the HOMO–LUMO transition, the energy of the CT state can be obtained from quasiparticle energies for a PEN HOMO, a C_60_ LUMO, and an e–h interaction:

(4) $$E_{CT}=\epsilon_{GW,L}^{I}-\epsilon_{GW,H}^{J}-U$$

Within the GW+BSE [23], *U* contains the exchange interaction and e–h screened Coulomb interaction for singlet excited states. However, we note that exchange interaction are negligibly small for intermolecular CT excitations [12]. Therefore, in this study, the e–h interaction is described as an e–h screened Coulomb interaction, $U=W_{II,JJ}$, where

(5) $$W_{II,JJ}=\int dr_{1}\int dr_{2}\psi_{H}^{I}\left(r_{1}\right)\psi_{H}^{I*}W\left(r_{1},r_{2}\right)\psi_{L}^{J}\left(r_{2}\right)\psi_{L}^{J*}\left(r_{2}\right),$$

where *W* is the statically screened Coulomb potential. If the exchange interactions are neglected, the energies of singlet CT states are identical to those of triplet CT states.

A delocalized CT state is composed of a delocalized electron and a delocalized hole, as represented in Figure 1. Such a delocalized CT state can be written as the superposition of multiple localized CT states, $|\Psi \rangle =\sum_{I,J}C_{I,J}|e_{I}h_{J}\rangle$, where $|e_{I}h_{J}\rangle$ denotes a localized CT state comprising the electron in an *I*th C_60_ molecule and the hole in a *J*th PEN molecule. The coefficients $C_{I,J}$ can be obtained by diagonalizing the following Hamiltonian.

(6) $$\langle e_{I}h_{J}\left|H\right|e_{K}h_{L}\rangle =\delta_{JL}H_{IK}^{e}-\delta_{IK}H_{JL}^{h}-W_{IK,JL}.$$

Here, *I* and *K* run over C_60_ molecules, while *J* and *L* run over PEN molecules. For simplicity, we drop the orbital indexes, and the HOMO of PEN molecules and the three degenerate LUMOs of C_60_ molecules were used in the excited-state Hamiltonian. Equation (6) corresponds to the excited-state Hamiltonian within Tamm-Dancoff approximation of the GW+BSE, where the configuration state functions describing the localized CT states are included only as basis functions for the Hamiltonian. In our calculations, intramolecular excited states (or exciton states) were not treated; thus, hybridization of the exciton states and CT states was not considered.

Here, we briefly compare the present method with earlier excited-state calculations in the FMO method [40,43,50,51,52]. In earlier FMO methods, excited states for a fragment monomer is obtained in the presence of electrostatic potential of other fragment which are determined for the electronic ground state. This scheme assumes that the electronic excitation of a fragment monomer does not have influence on the charge densities of the surrounding fragments. In other words, although the electrostatic contribution of polarization energy can be considered, the induction contribution cannot be incorporated into the excited states. The neglect of excited-state polarization effect resulted in the overestimation of charge-separated states [15]. In contrast, the present FMO-GW method can appropriately describe induction contribution of polarization energy of CT states, in which the screening of e–h interaction by can be considered.

## 3. Results and Discussion

### 3.1. Energy Levels at the Interface

**Figure A1:**
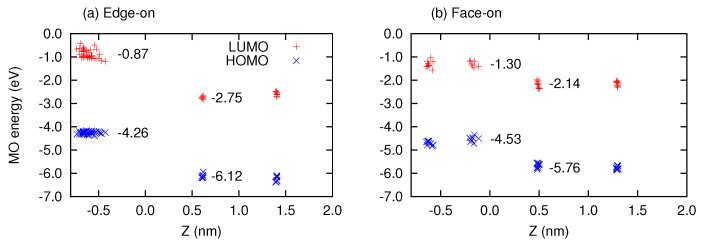
The energies of HOMOs and LUMOs in the (**a**) edge-on and (**b**) face-on interfaces along the direction perpendicular to the interfaces. The mean values of the HOMOs and LUMOs in the vicinity of the interface are explicitly shown. Reproduced with permission from ref. [21]. Copyright 2019, American Institute of Physics.

In this section, we consider the energy levels at the edge-on and the face-on interfaces. We being by presenting the HOMO and LUMO quasiparticle energies, which represent the energy levels for the localized charge carriers. The HOMO and LUMO for a PEN and for a C_60_ molecule in the gas phase (an isolated PEN or a C_60_ molecule) and those in the edge-on and face-on interfaces are summarized in Table 1. In the interface structures, the HOMO or LUMO energies were obtained as the average of the PEN or C_60_ molecules in the vicinity of the interface. HOMO and LUMO energies with respect to the direction perpendicular to the interface are provided in Figure A1. The HOMO/LUMO energies of isolated molecules are −5.56/−0.74 eV for PEN and −6.74/−1.84 eV for C_60_. Here, the energy difference between the PEN HOMO and C_60_ LUMO governs the CT energy. In a gas phase, the energy difference between the C_60_ LUMO and PEN HOMO is 3.72 eV. Solid-state effects have substantial influence on the HOMO and LUMO energies. Here, the corresponding gaps in the interface structures, which include both electrostatic and induction contributions (“E+I” in Table 1), were obtained as 1.51 and 2.39 eV for the edge-on and face-on structures, respectively. The C_60_ LUMO–PEN HOMO gap value is significantly lower than that of the gas phase. Moreover, the interfacial morphology have considerable influences on the MO energy levels, and the face-on interface exhibits the gap 0.84 eV larger than that of the edge-on interface.

**Figure 3:**
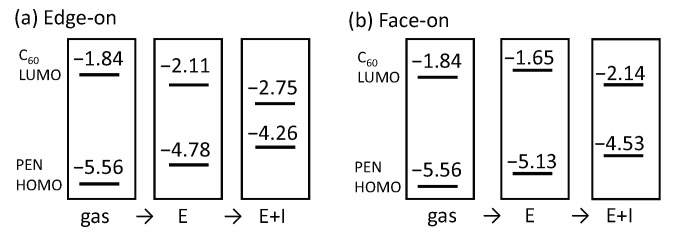
Evolution of C_60_ LUMO and PEN HOMO levels for the (**a**) edge-on and (**b**) face-on interfaces: MO energies in a gas phase, those with electrostatic (E) contribution in the interfaces, and those with both electrostatic and induction (E+I) contributions.

To understand the dependence of interfacial orientations, we investigate the MO energy changes between the gas and interface structures in terms of the electrostatic and induction contributions of the polarization energy. In the PEN/C_60_ structures, the quadrupole moments of PEN molecules are predominantly responsible for the electrostatic contribution. Figure 3 schematically represents the MO energies in a gas phase, those including the electrostatic contribution, and those with both electrostatic and induction contributions. We found that the electrostatic components are responsible for the intefacial morphology dependence. The PEN molecules feature permanent quadrupolar charge distributions with partial positive charges in the plane and partial negative charges above and below the plane; these charge distributions lower (stablize) the C_60_ LUMO levels in the edge-on interface, but raise (destablize) the C_60_ LUMO levels in the face-on interfaces, respectively. In contrast to the electrostatic effects, the induction effects have similar influences in both interfaces and tend to reduce the C_60_ LUMO–PEN HOMO gap. Those results are consistent with earlier suggestions that the electrostatic contributions are responsible for the dependence on interfacial orientations [35,53,54], while the induction effects are insensitive to interfacial orientations.

Next, we consider the energy difference between the PEN LUMO and C_60_ LUMO. The driving energy for the charge separation is often estimated as the offset between donor and acceptor LUMOs (or HOMOs). The solid-state polarization effects also show substantial influence on the LUMO offset. The LUMO energy offset in a gas phase is 1.10 eV, while those in the edge-on and the face-on interfaces are 1.84 and 1.01 eV, respectively. The evolution of the LUMO energy offset in the edge-on interface are 1.10 (gas), 1.92 (E), and 1.88 eV (E+I). On the other hand, the evolution of the LUMO energy offset in the face-on interface are 1.10 (gas) 1.01 (E) and 0.84 eV (E+I). As with the PEN HOMO–C_60_ LUMO gap, the morphology dependence is predominantly determined by the electrostatic contribution; the electrostatic effects increase and decrease the LUMO offsets in the edge-on and face-on interfaces, respectively. The effects of the induction contribution on the LUMO offset is slightly more pronounced in the face-on than in the edge-on structures. However, these results may be partly ascribed to the finite system size in the FMO-GW calculations.

**Figure 4:**
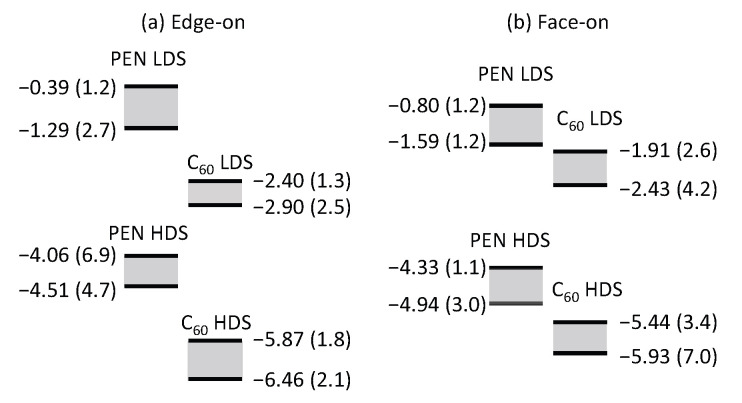
Schematic energy level diagrams for HOMO-derived states and LUMO-derived states in the PEN/C_60_ interfaces in the (**a**) edge-on and (**b**) face-on interfaces, with energies shown in unit of eV and IPRs provided in parentheses.

**Figure A2:**
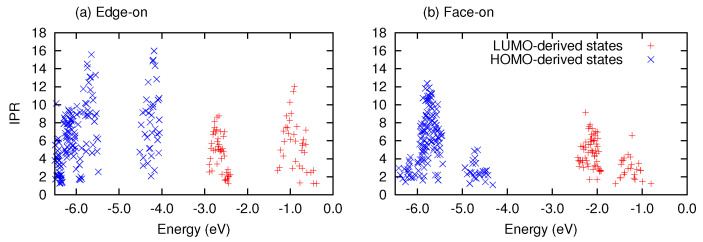
The IPRs for the one-electron orbitals in the (**a**) edge-on and (**b**) face-on interfaces.

Having investigated the MO energy levels, we turn to the energy levels of delocalized orbitals. In a disordered molecular aggregate, delocalized and localized orbitals can coexist. The extent of the delocalization can be characterized by the inverse participation ratio (IPR) [55,56], which are provided in Figure A2. Although the PEN HOMO-1 orbitals are slightly hybridized with C_60_ HOMOs, most orbitals are distributed within PEN molecules or within C_60_ molecules. Therefore, we considered the lowest HOMO-derived states (HDSs) and LUMO-derived states (LDSs) within the PEN molecules or within the C_60_ molecules. The energies and IPRs of highest and lowest HDSs and LDSs are schematically shown in Figure 4. The gap between the lowest C_60_ LDS and highest PEN HDS are 1.16 eV in the edge-on interface and 1.99 eV in the face-on interface. These gaps are smaller than the corresponding gaps defined by the MOs, showing the importance of orbital delocalization in the determination of energy levels. The IPRs in the edge-on structure are larger than those in the face-on structure. The PEN stacking structures are more disordered in the face-on interface, suppressing charge delocalization.

We now compare our calculations with the results from a previous experimental study. Nishi et al. determined the energy levels of edge-on and face-on orientations of PEN/C_60_ bilayer heterojunctions using ultraviolet photoelectron spectroscopy (UPS) [31]. The energies of PEN HDSs, which were estimated from the UPS spectra, are 4.82 and 5.45 eV relative to the vacuum level in the edge-on and the face-on interfaces, respectively. Those values correspond to the negative of the energy of the highest PEN HDSs in our calculations. Although the calculated energy levels (4.06 eV in the edge-on and 4.33 eV in the face-on) are underestimated because of the relatively small basis function in our GW calculations, the dependence of interfacial orientations is reasonably well reproduced. Nishi et al. also estimated the energy gap between PEN HDS and C_60_ LDS as the 0.75 eV in the edge-on and 1.50 eV in the face-on interfaces; these values are noticeably smaller than our estimations (1.16 eV in the edge-on and 1.99 eV in the face-on interfaces). We note that Nishi et al. assumed the fundamental gap of PEN and that of C_60_ in the interface are same as those in the bulk phase. However, due to the structure disorder inherent in the interface, the one-electron orbitals in the vicinity of the interfaces would be more localized than in the bulk phase. Hence, in the vicinity of the interface, the fundamental gap would be larger than that in the bulk phase. Indeed, their reported gap of 0.75 eV in the edge-on interface is smaller than the CT absorption peak extracted from EQE measurement [32,33]. The inconsistency of this finding becomes clear when we consider that the CT energy (e.g., $\epsilon_{GW,L}^{I}-\epsilon_{GW,H}^{J}-U$) is generally smaller than the corresponding fundamental gap (e.g., $\epsilon_{GW,L}^{I}-\epsilon_{GW,H}^{J}$).

### 3.2. Interfacial CT States

In this section, we consider the CT states across the PEN/C60 interfaces. First, we investigate the electrostatic and induction contributions of the polarization energies of localized CT states. For MO energies, the polarization energy refers to the interaction between a charge carrier and the surronding molecules. On the other hand, the polarization energy of a CT state represents the interaction between the e–h pair and the surrounding molecules. The electrostatic contribution arises from the permanent multipole moments of the surrounding molecules, while the induction contribution is dominated by dipole moments of surrounding molecules that are induced by the formation of the CT state. It is expected that the induction effects increase with increasing e–h separation, since the dipole moment of the e–h pair increases.

**Figure A3:**
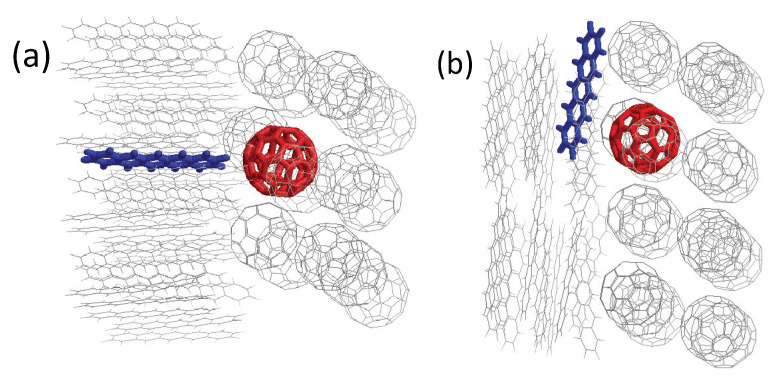
Visualization of representative CT states in the (**a**) edge-on and (**b**) face-on interfaces. The molecules in which an electron and a hole are localized, are represented in red and blue colors, respectively. Reproduced with permission from ref. [21]. Copyright 2019, American Institute of Physics.

Here, the localized CT states comprising nearest-neighbor PEN–C_60_ pairs are considered to be representative states. Visualization of these CT states are provided in Figure A3. The results for these representative CT states are shown in Table 2, in which the CT energy ($E_{CT}$) is decomposed into the C_60_ LUMO-PEN HOMO gap (Δϵ) and the e–h interaction. The results for the gas phase were obtained from the corresponding FMO-GW+BSE calculation for the isolated PEN–C_60_ pair without any surrounding molecules. In a gas phase, the $E_{CT}$ of the edge-on orientation is higher than that of the face-on orientation, as a result of lower e–h interaction of the edge-on. The e–h distances that are defined as the center-of-mass distance between the electron and hole distributions are 1.3 and 0.9 nm for these edge-on and face-on pairs, respectively, resulting in the lower e–h interaction for the edge-on pair. Solid-state polarization effects also have a considerable impact on CT energies. As with the MO energies, the electrostatic effects reduce C_60_ LUMO-PEN HOMO gap of the edge-on and increase the gap of face-on pairs, without altering the e–h interaction. By contrast, the induction effects reduce both the C_60_ LUMO-PEN HOMO gaps and the e–h interactions, lowering the CT energies by 0.79 and 0.53 eV for the edge-on and face-on pairs, respectively. Because of the larger e–h separation of the edge-on pair, as mentioned above, the CT state of the edge-on pair is more stablized by the induction interaction than the face-on pair.

**Figure 5:**
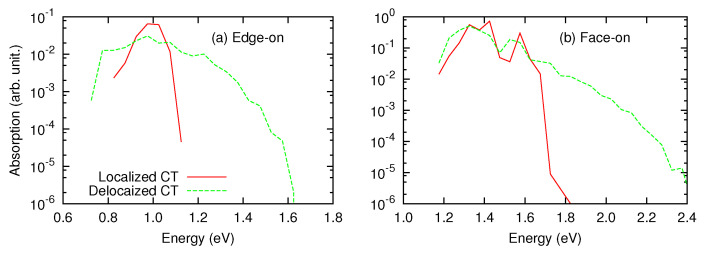
Absorption spectra of localized and delocalized CT states in the (**a**) edge-on and (**b**) face-on interfaces.

We next turn to the delocalized CT states. The diagonalization of the excited-state Hamiltonian yielded the CT-state manifold containing more than 2000 states. Here, we characterize these excited state in terms of the oscillator strength and spatial extent of electron or hole distributions. The absorption spectra for the edge-on and face-on structures are shown in Figure 5, with corresponding results of localized CT states (omitting charge delocalization effects). In the edge-on and face-on interface structures, the peak positions are approximately located in the 0.98 and 1.33 eV, respectively. Although the delocalization effects have minor effects on the peak positions, they substantially alter the lineshape of the absorption spectra. In particular, the absorption spectra became broader via the delocalization effects, with absorption tails in higher-energy regions. The calculated absorption spectra can be compared with the EQE spectra. Lin et al., [33] investigated the dependence of CT energies of PEN/C_60_ interfaces under different morphological conditions. According to their EQE spectra, the 0–0 transition energies of CT states were extracted in the range 0.97–1.09 eV, while that of 1:8 PEN/C_60_ bulk heterojunction on indium tin oxide is 1.33 eV. In most of their experimental conditions, PEN molecules adopt a herringbone stacking configuration, in which the PEN long axis is oriented nearly perpendicular to the interface [33], corresponding to the edge-on orientation in the present study. Therefore, the calculated peak position of the edge-on structure (0.98 eV) can be compared well to their experimental results. We found that the peak position of the face-on interface is in reasonable agreement with their result for 1:8 PEN/C_60_ bulk heterojunction on ITO. This agreement indicates that the CT absorption of the 1:8 PEN/C_60_ bulk heterojunction on ITO is dominated by the face-on oriented PEN/C_60_ pairs.

**Figure 6:**
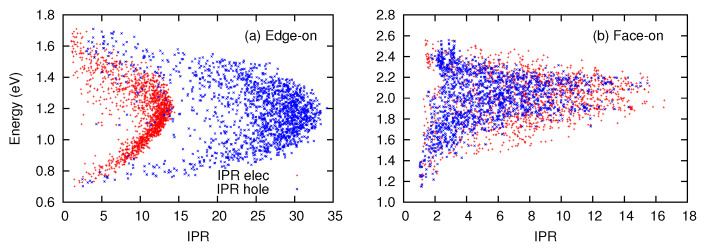
Inverse participation ratios (IPRs) of electron and hole distributions constituting CT states in the (**a**) edge-on and (**b**) face-on interfaces.

To examine the CT states in greater detail, we examine the spatial extents of the electron and hole distributions that constitute a CT state. In Figure 6, the electron and hole IPRs are shown for the edge-on and the face-on interfaces. Despite the similar spatial extents of the electron distributions in both interfaces, the hole IPRs in the edge-on interface are larger than those in the face-on interface. The molecular orientations at the interface are more disordered in the face-on interface than in the edge-on interface, tending to cause localization of the charge distribution. In addition, charge delocalization of CT states can be more enhanced in the direction parallel to the interface than in the direction perpendicular to the interface: In general, the extent of delocalization is more enhanced if composite localized states have similar energies. In the face-on configuration, the stacking direction of PEN molecules runs perpendicular to the interface. However, the hole delocalization along the stacks increases the excitation energy, because the delocalized CT state becomes composed of long-range CT states of higher energy. That is, hole delocalization in the face-on configuration is suppressed by the energy variations among CT states. In contrast, the PEN stacks run parallel to the interface in the edge-on configuration, and the delocalization over the stack does not increase the e–h separation. The CT states in the edge-on interface can be delocalized without being mixed with higher-energy states.

**Figure 7:**
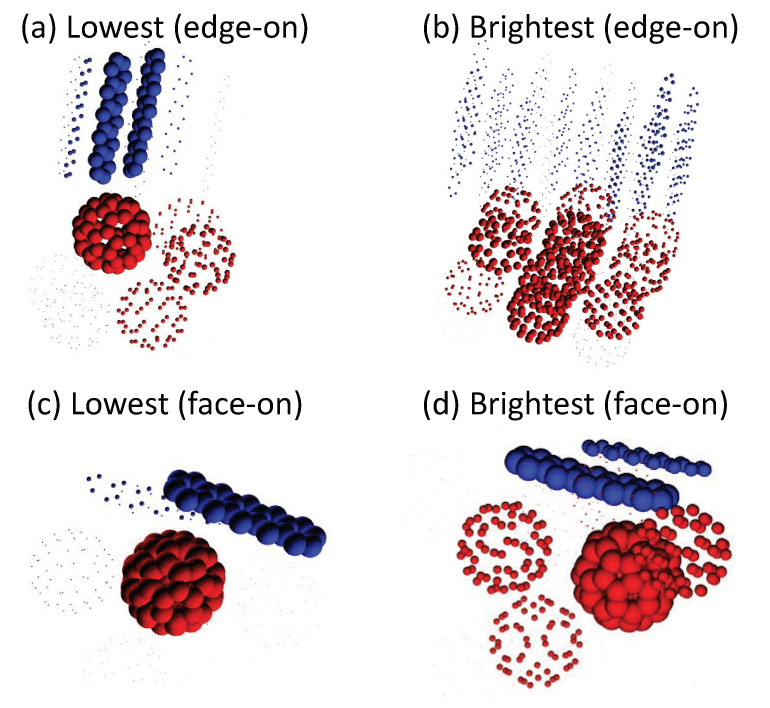
Visualization of the (**a**) lowest and (**b**) brightest CT state in the edge-on interface, and the (**c**) lowest and (**d**) brightest CT state in the face-on interface, where positive and negative charge distributions are represented in blue and red colors, respectively.

Here, the spatial extents of the electron and hole distributions of the representative delocalized CT states are visualized in Figure 7. The lowest CT state among the CT-state manifold in the edge-on or the face-on interfaces is considered. Furthermore, the CT state whose oscillator strength is largest among the CT-state manifold is shown to be the “brightest” CT state. The energies and wave function properties of the lowest and brightest CT states are shown in Table 3. In the edge-on interface, the lowest CT state is relatively localized, while the brightest CT state is significantly delocalized, with electron and hole IPRs of 9.92 and 25.73, respectively. In the face-on interface, by contrast, both the lowest and brightest CT states are relatively localized, with both electron and hole IPRs ranging from 1 to 2. As discussed above, hole delocalization in the face-on interface increases the e–h separation and thus reduces the oscillator strength; such the delocalized CT states appear in the higher-energy region with smaller absorption intensities. It has been discussed that the low-lying CT states are dominantly responsible for the device operation [4]. The present results indicate that the edge-on interface, in which *π*-*π* stacking structures run parallel to the interface, allows for the charge delocalization in low-energy CT states and thus enables efficient charge separation.

### 3.3. Estimation of Charge Transfer Exciton Binding Energy

The charge transfer exciton binding energy refers to the energy necessary to dissociate the bound e–h pair into free charge carriers and thus is one of most important factors in charge separation. In the excited-state calculation within GW+BSE, the excitation energy can be decomposed into the orbital–energy difference and the e–h interaction. Thus, it is straightforward to define the exciton binding energy as the e–h interaction (*U*). On the other hand, exciton binding energy is experimentally estimated as the energy difference between the fundamental gap and optical gap [58,59]. These different estimations can result in considerable variations in exciton binding energy. According to our calculation, the fundamental gap, obtained as the difference between the highest HDS and the lowest LDS, are 1.16 eV in the edge-on and 1.89 eV in the face-on interfaces. If CT energies are assigned according to the peak positions of their absorption spectra (0.98 eV in the edge-on and 1.33 eV in the face-on), then estimated exciton binding energies in the edge-on and the face-on interfaces are 0.18 and 0.56 eV, respectively. These values are noticeably smaller than values from the e–h interaction; for example, *U* of the brightest CT states in the edge-on and the face-on are 0.58 and 0.90 eV, respectively. The difference between the two estimates comes from the larger values of the orbital-energy difference in excitation energy, compared to the fundamental gap, which are defined as the difference between the highest HDS and the lowest LDS.

## 4. Conclusions

We investigated the energy levels and CT states of the edge-on and face-on orientations of the PEN/C_60_ interfaces. The applications of the fragment-based GW/BSE method allowed for detailed analyses of the polarization and charge delocalization effects in the electronic states. Calculated energy levels and CT energies are in reasonable agreement with the experimental estimates. The interfacial orientation have considerable effects on the energy levels and CT states. The dependence of the energy levels on interfacial morphology is predominantly determined by the electrostatic contribution of quadropole polarization energy, while the induction dipolar contribution have similar effects in both the edge-on and the face-on interfaces. In the edge-on interface, the delocalized CT state can exist as low-lying CT states in the CT-state manifold. By contrast, low-lying CT states in the face-on interface are relatively localized. In previous studies, energy levels of donor/acceptor interfaces have often been estimated from cyclic voltammetry measurements in solution or UPS spectra of donor or acceptor thin films. However, our results indicate that careful assessments are necessary to determine the actual electronic states for specific donor/acceptor morphologies. Our calculations provides the microscopic insight into the relationship between interfacial morphologies and electronic states, as well as into the roles of polarization and delocalization effects.

## Figures and Tables

**Table 1:** HOMO and LUMO energies for single PEN and C_60_ molecules in the gas phase, those including the electrostatic contribution (E) in the interfaces, and those including both electrostatic and induction (E+I) contributions in the interfaces. In the interface structures, HOMOs and LUMOs were obtained as the average of the PEN or C_60_ molecules in the vicinity of the interfaces.

		PEN	C_60_
gas ^(*a*)^	LUMO	−0.74	−1.84
	HOMO	−5.56	−6.74
Edge-on (E)	LUMO	−0.28	−2.12
	HOMO	−4.78	−6.69
Edge-on (E+I)	LUMO	−0.87	−2.75
	HOMO	−4.26	−6.12
Face-on (E)	LUMO	−0.63	−1.64
	HOMO	−5.13	−6.22
Face-on (E+I)	LUMO	−1.30	−2.14
	HOMO	−4.53	−5.76

^(*a*)^ The molecular geometry was optimized at the B3LYP/6-31G** level using Gaussian 16 [57].

**Table 2:** The energies $\left(E_{CT}\right)$, PEN HOMO-C_60_ LUMO gaps (Δϵ), and e–h Coulomb interaction (U) in eV for the representative localized CT states in gas phase, those including the electrostatic (E) contribution, and those with the electrostatic and induction (E+I) contributions in the edge-on and the face-on interfaces.

		$E_{\mathrm{CT}}$	Δϵ	*U*
Edge-on	gas ^(*a*)^	1.92	3.18	1.27
	E	1.70	2.96	1.26
	E+I	0.91	1.58	0.68
Face-on	gas ^(*a*)^	1.50	3.11	1.61
	E	1.72	3.33	1.61
	E+I	1.19	1.99	0.80

^(*a*)^ Results for the isolated PEN-C_60_ pairs without any surrounding molecules.

**Table 3:** The energies in unit of eV and wave function properties of the lowest and brightest (largest oscillator strength) CT states in the edge-on and the face-on interfaces.

		$E_{\mathrm{CT}}$	Δϵ	*U*	*R_eh_* (nm)	IPR Elec	IPR Hole	OS
Edge-on	Lowest	0.70	1.41	0.71	1.35	1.50	2.55	8.9 × 10^−5^
	Brightest ^(*a*)^	0.99	1.57	0.58	1.53	9.92	25.73	2.3 × 10^−3^
Face-on	Lowest	1.15	1.96	0.81	0.84	1.09	1.14	0.03
	Brightest ^(*a*)^	1.40	2.30	0.90	0.76	1.98	1.32	0.29

^(*a*)^ The CT state whose oscillator strength is largest among the CT-state manifold.

## Abbreviations

The following abbreviations are used in this manuscript:

BSE	Bethe-Salpeter equation
COHSEX	Coulomb-hole plus screened exchange
DFT	density functional theory
EQE	external quantum efficiency
FMO	fragment molecular orbital
TDDFT	time-dependent density functional theory
